# The relationship between different dialysis methods and septicemia: a systematic review and meta-analysis

**DOI:** 10.1080/0886022X.2020.1776733

**Published:** 2020-06-18

**Authors:** Zhu Liduzi Jiesisibieke, Songyu Zhang, Ching-Wen Chien, Tao-Hsin Tung

**Affiliations:** Institute for Hospital Management, Tsing Hua University, Shenzhen, Guangdong, China; b Maoming People’s Hospital, Maoming, Guangdong, China; c Department of Medical Research and Education, Cheng Hsin General Hospital, Taipei, Taiwan

Dear Editor,

Kidney failure is a general term for heterogeneous disorders affecting kidney structure and function, which becomes a worldwide problems due to its frequency and high costs [1,2], and a large number of them are in need for several treatments including dialysis [3]. The patients undergo dialysis is likely to have infectious complications that contribute to morbidity and mortality [4]. Evidence-based studies proved that access-related infections (ARIs) are the main source of morbidity, mortality and additional health care costs in hemodialysis (HD) and peritoneal dialysis (PD) patients [5]. Although international guidelines recommending the avoidance of catheters for hemodialysis access, hospital admissions for vascular ARIs have increased significantly in the last decade. Whether different dialysis modes are associated with septicemia is an important clinical issue worthy to study. However, the impact of different dialysis methods on septicemia is still unclear. The purpose of this study is to assess the respective risk estimates of sepsis in patients with different dialysis.

**Figure 1.**
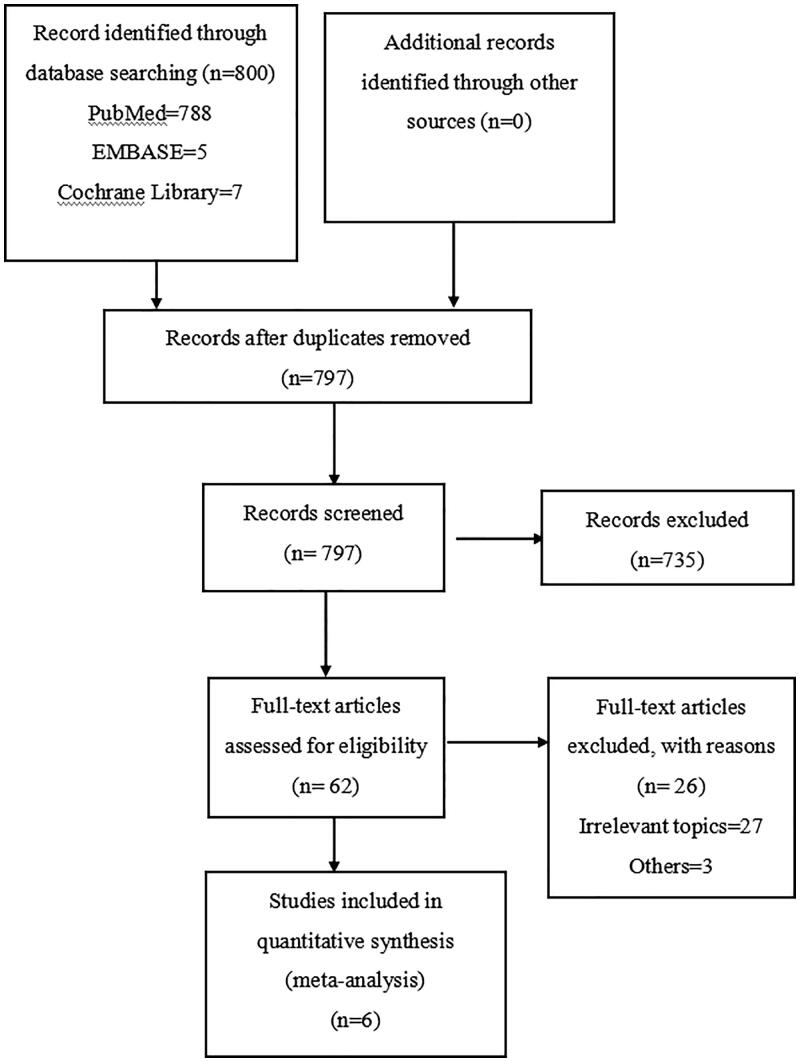
PRISMA study flow chart.

Electronic searches of the Cochrane Library, PubMed, and EMBASE for relevant studies from inception to 31 October 2019 were conducted in this study. The search string was ‘(hemodialysis OR peritoneal dialysis) AND (septicemia OR blood poisoning OR hematosepsis)’ with no limitations on language. We included studies that met the following criteria: 1. the study design was cohort study or longitudinal study; 2. the exposure group was patients treated with HD and the control group was patients treated with PD; 3.the article reported the risk for septicemia or bacteremia or blood poisoning or hematosepsis. We conducted this study according to the Preferred Reporting Items for Systematic Reviews and Meta-Analyses (PRISMA) guidelines (Figure 1). No hand searching was performed. All selected studies were low-to-moderate risk using ROBINS-I approach (Table 1). The summary of findings and the GRADE assessment for each outcome is presented in Table 2. In this study, we used the Review Manager 5.3 (The Nordic Cochrane Center, The Cochrane Collaboration, 2014).

**Table 1.** Risk of bias assessment using ROBINS-I.

Author	Types of research	Pre-intervention		At intervention	Post-intervention				Total
		Bias due to confounding	Bias in selection of participants into study	Bias in classification of interventions	Bias due to de*via*tions from intended interventions	Bias due to missing data	Bias in measurement of outcomes	Bias in selection of the reported outcomes	Total bias
Aslam et al. [6]	Prospective cohort study	Low risk	Low risk	Low risk	Low risk	Low risk	Moderate risk	Low risk	Low risk
Foley et al. [7]	Cohort study	Low risk	Moderate risk	Low risk	Low risk	Low risk	Moderate risk	Low risk	Low risk
Jin et al. [8]	Retrospective study	Moderate risk	Low risk	Low risk	Low risk	Low risk	Moderate risk	Moderate risk	Moderate risk
Koch et al. [9]	Observational cohort study	Moderate risk	Low risk	Low risk	Low risk	Low risk	Moderate risk	Low risk	Low risk
Powe et al. [10]	Longitudinal cohort study	Moderate risk	Low risk	Moderate risk	Low risk	Low risk	Moderate risk	Low risk	Moderate risk
Wang et al. [11]	Retrospective cohort study	Low risk	Low risk	Low risk	Moderate risk	Low risk	Moderate risk	Low risk	Low risk

**Table 2.** GRADE summary of findings.

Relationship between different dialysis methods and septicemia						
Patient or population: patients treated with dialysis Setting: China, Germany, United States Comparison: hemodialysis and peritoneal dialysis						
Outcomes	Anticipated absolute effects^a^ (95% CI)		Relative effect (95% CI)	No. of participants (studies)	Quality Of the evidence (GRADE)	Comments
	Risk in control	Risk in experiment				
Risk of septicemia	80 per 1000	174 per 1000	OR 2.45 (1.53–3.90)	349277 (6 observational studies)	⨁⨁⨁◯ Moderate	

^a^The risk in the intervention group (and its 95% confidence interval) is based on the assumed risk in the comparison group and the relative effect of the intervention (and its 95% CI). CI: Confidence interval; OR: Odds ratio

GRADE Working Group grades of evidence – High quality: We are very confident that the true effect lies close to that of the estimate of the effect. Moderate quality: We are moderately confident in the effect estimate: The true effect is likely to be close to the estimate of the effect, but there is a possibility that it is substantially different. Low quality: Our confidence in the effect estimate is limited: The true effect may be substantially different from the estimate of the effect. Very low quality: We have very little confidence in the effect estimate: The true effect is likely to be substantially different from the estimate of effect.

**Figure 2.**
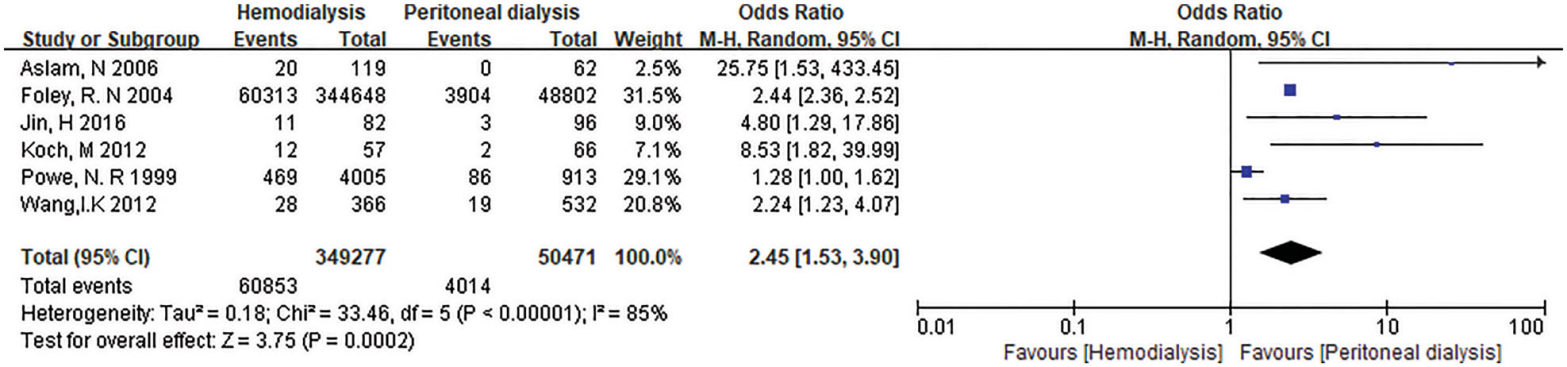
Odds of Septicemia in patients treated with HD or PD. CI: confidence interval; SE: standard error.

This study identified 797 articles after removing duplicates. Eventually, 6 publications with a total of 399,748 study subjects met our inclusion criteria. Compared to PD group HD was significantly associated with septicemia (OR = 2.45, 95%CI: 1.53–3.90; Figure 2).

All of the six studies proved that HD patients had a significantly higher proportion of bacteremia. The study by Wang et al. [11] included 366 patients treated with HD, 532 patients treated with PD. The study by Koch et al. [9] included 57 patients treated with HD and 66 patients treated with PD. The study by Aslam et al. [6] included 119 patients treated with HD and 62 patients treated with PD. The study by Powe et al. [10] included 4005 patients treated with HD, 913 patients treated with PD. The study by Foley et al. [7] included 344,648 patients treated with HD, 48,802 patients treated with PD. Jin et al. [8] included 82 patients treated with HD, 96 patients treated with PD. All these study provide odds ratio (OR) or risk ratio (RR). Previous study estimated that septicemia is associated with organ dysfunction, hypoperfusion or hypotension [12]. Pre-onset factors have a strong impact on the outcome of sepsis, thereby changing the disease process and treatment [13]. The pre-onset factors include the presence of comorbidities such as diabetes, and repetitive exposure to pathogens during hemodialysis [14].

Previous longitudinal study indicated that among 4918 ESRD patients found that sepsis was higher for HD (11.7%) than for PD patients (9.4%) [10]. Whether PD or HD treatment for ESRD patients is an important medical decision making for patients considering cost, quality of life and survival. Our findings are important for reducing the morbidity of septicemia among ESRD patients. As result of that, medical staff could tell the patients the different outcomes of PD and HD, and let them do the choices.

This study has some limitations. Firstly, due to the selected studies from the databases which could be search may be not sufficient, the relative lower statistical power with insufficient sample sizes is inevitable. Secondly, the hypothesis of normal distribution for random effects is against the principle of randomization in the inferential statistics [15]. Thirdly, it is difficult to do subgroup analyses according to demographic variables such as sex, age, and concurrent comorbidity because the selected studies did not include enough information. Future studies should be conducted to explore outcomes and confirm whether HD is an independent risk factor for septicemia. Finally, I^2^ test seeks to determine whether there are real differences according to the findings of the selected studies, that is, heterogeneity, or whether the variation in results is reconcilable with chance alone, that is, homogeneity. I^2^ values of 0–24.9%, 25–49.9%, 50–74.9%, and 75–100% were viewed as none, low, moderate, and high heterogeneity, respectively. In this study, we used the random-effect model when I^2^ statistics was 85% more than 50%. However, we aggregate studies that are different methodologies, heterogeneity is still inevitable in the meta-analysis.

In conclusion, our study suggests a relationship between HD and septicemia among ESRD population. To further examine this finding and establish a stronger temporality, more large-scale prospective studies are warranted to provide more information about the details of the association between different dialysis treatments and septicemia. An increased rate of septicemia occurs in HD patients and clinicians should be aware of this possibility.
